# An Appearance-Based Tracking Algorithm for Aerial Search and Rescue Purposes †

**DOI:** 10.3390/s19030652

**Published:** 2019-02-05

**Authors:** Abdulla Al-Kaff, María José Gómez-Silva, Francisco Miguel Moreno, Arturo de la Escalera, José María Armingol

**Affiliations:** Intelligent Systems Lab (LSI), Universidad Carlos III de Madrid, Avnd. de la Universidad 30, 28911 Madrid, Spain; magomezs@ing.uc3m.es (M.J.G.-S.); franmore@ing.uc3m.es (F.M.M.); escalera@ing.uc3m.es (A.d.l.E.); armingol@ing.uc3m.es (J.M.A.)

**Keywords:** multi-object tracking, UAV, rescue, reactive control

## Abstract

The automation of the Wilderness Search and Rescue (WiSAR) task aims for high levels of understanding of various scenery. In addition, working in unfriendly and complex environments may cause a time delay in the operation and consequently put human lives at stake. In order to address this problem, Unmanned Aerial Vehicles (UAVs), which provide potential support to the conventional methods, are used. These vehicles are provided with reliable human detection and tracking algorithms; in order to be able to find and track the bodies of the victims in complex environments, and a robust control system to maintain safe distances from the detected bodies. In this paper, a human detection based on the color and depth data captured from onboard sensors is proposed. Moreover, the proposal of computing data association from the skeleton pose and a visual appearance measurement allows the tracking of multiple people with invariance to the scale, translation and rotation of the point of view with respect to the target objects. The system has been validated with real and simulation experiments, and the obtained results show the ability to track multiple individuals even after long-term disappearances. Furthermore, the simulations present the robustness of the implemented reactive control system as a promising tool for assisting the pilot to perform approaching maneuvers in a safe and smooth manner.

## 1. Introduction

The recent and continuously increasing research in the field of Unmanned Aerial Vehicles (UAVs) has boosted them as suitable platforms for carrying sensors and computer systems in order to perform advanced tasks, such as terrain thematic and topographic mapping [1,2,3]; exploration of unreachable areas like islands [4], rivers [5], forests [6] or oceans [7]; for surveillance purposes [8,9]; for traffic monitoring [10], including the estimation of the traffic flow behavior [11], and traffic speed [12]; and search and rescue operations after disasters [13,14,15].

Wilderness Search and Rescue (WiSAR) missions place special requirements on small aerial robotic systems since it is the process of finding and assisting individuals who are lost in remote wilderness areas. UAVs provide potential support to human task forces in situation recognition, by performing automatic perception and complex maneuvers in these environments, reducing the operational time and cost [16].

A stable communication cannot be guaranteed in such environments, and because of that, a high level of autonomy of the systems is required. However, the limited availability of computing resources and low-weight sensors for UAVs generate a great challenge in achieving such a level of autonomy not only in the control stage but also in the perception one.

For that reason, computer vision plays an essential role in the automation of the search task, thanks to the development of detection and tracking algorithms. Some methods allow the tracking of one user-selected object, like that presented in [17], and others even track multiple people and vehicles [18,19,20].

To our knowledge, we present the first known work applying Multi-object visual tracking from aerial images for search and rescue purposes. The nonexistence of labeled rescue datasets prevents any possible comparison in a benchmark datasets. With the aim of proving the potential application of our method in real scenarios, a modest set of sequences, as well as its ground truth, have been generated.

In this paper, we propose a detection and multi-object tracking algorithm based on color and depth data streams provided by a low-weight and low-cost sensor, unlike another branch of methods based on thermal technologies [21,22].

A traditional tracking approach based on the model of the trajectories [23,24] through Bayesian probabilities [25] is not applicable in the case of sequences captured from a fast-motion platform, where the position of the agents drastically changes between consecutive frames and they even temporarily disappear. In order to perform the tracking with these conditions, the proposed approach is mainly based on the visual appearance of the targets, which allows even the re-identification of the targets after long-term disappearances.

In addition, this paper discusses a set of possible approaches to obstacle avoidance that does not take into consideration the map knowledge [26]; therefore a case where information available to the UAV is purely local. The advantage of using local data is that it allows the UAV to adapt to the changing in the environment in a reactive manner in real-time. This type of control, that describes the cooperation of autonomous and manual control, is known as Semi-Automatic control.

Therefore, this paper presents a detection and tracking algorithm for rescuing victims in disaster environments using aerial images, aided by a semi-autonomous reactive control, whose main contributions are summarized as follows:

- A human detection stage based on color and depth data and the use of a Human Shape Validation Filter. This filter employs the human joints locations provided by a Convolutional Pose Machine (CPM) [27] to study the human skeleton shape of the found detections, avoiding the false positive ones.
- An automatic Multi-Object Tracking algorithm, which is invariant to a scale, translation and rotation of the point of view with respect to the target objects.
- A novel matching method based on pose and appearance similarity that is able to re-identify people after long term disappearances in the scene.
- A semi-autonomous reactive control is presented; to allow the pilot to approach the UAV to the detected object in a safe and smooth maneuver.

The remainder of this paper is organized as follows. Section 2 introduces some of the state-of-the-art techniques used. Section 3 explains a general description of the proposed system. In Section 4, the proposed human detection method and the Multi-Object Tracking algorithm are presented, followed by the description of the reactive control approach in Section 5. Section 6 discusses the experimental results. Finally, conclusions are summarized in Section 7.

## 2. Related Work

Computer vision with UAVs plays an essential role in a huge number of applications and tasks related to the UAVs [28]; especially in search and rescue operations [29], that is because of the performing photogrammetric data acquisition and offering many advantages for emergency response situations [30]. A vast amount of research has been developed in the research domains of sensing, perception and control, whose automation is essential for the WiSAR tasks.

### 2.1. Sensing

The literature presents a wide variety of sensors that can be used for search and rescue purposes. Using depth sensors to construct 3D models of the disaster scenes is common in many works; such as [31,32], which provides accurate distances information of the detected objects. In [33], a rescue assistance tool is presented, where a system based on the 3D modeling of the rubble piles is implemented in order to perform photometric analysis of 3D modeling of the disaster scenes and increase the ability to detect possible victims. With the aim of improving the 3D mapping result, a heterogeneous robot collaboration of UGV-UAV is presented in [34]. These collect observations in cluttered urban environments, where the robot team is able to map the environment while following predefined waypoints. First, the UGV builds the 3D map of the environment using a LiDAR, thereafter, the UAV performs the data gathering process. Moreover, the UAV estimates its location by detecting and tracking the UGV. However, if the UAV loses the sight of the UGV, it stops the progress and communicates with the UGV to start localizing it.

Moreover, there are many works based on thermal technologies, such as the presented by [21] and [22]. A system for fire and human detection using thermal and color aerial images is presented in [35]. In this system, an MSER blob detector with a color based descriptor is applied to the thermal and RGB images; for detection purposes. The system showed promising work to be used for wildfire situations, however, the authors did not present real experiments to validate their system.

In [36], pedestrian detection from aerial images with the estimation of its distance using monocular camera is presented. In this work, the pedestrian detection is based on the Histogram Oriented Gradients (HOG) descriptor. Thereafter, by using a three image sequence, the distance to the pedestrian is estimated. However, two constraints should be considered in this system; first, the UAV location must be always known using other sensors, such as GPS or WiFi, and second, the system is limited to detect pedestrian within a distance of 11 m.

Recently, the use of RGB-D sensors, as it is proposed by this work, is increasingly becoming more common. Perez-Grau et al. presented a vision-based system for semi-autonomous teleoperation purposes [37], with a visual odometry approach based on an RGB-D sensor. This provides information about the UAV traveled distance within an interval of time. Meanwhile, the manual commands are used to correct the drifts generated by the visual odometry along the time or to perform collision avoidance maneuvers.

### 2.2. Perception

In WiSAR tasks, not only is obstacles detection needed but the tracking of the individuals to rescue is also required, in order to get an accurate knowledge of the number and situation of the humans in the scene. Multi-object tracking (MOT) consists of automatically finding the location of multiple targets from their visual measurements and correctly associating their identities in a video sequence. The most common architecture for MOT algorithms involves object detection and a data association stage to match each object with its identity. This type of approaches has been commonly encompassed under the well-known “tracking-by-detection” paradigm.

The literature provides a wide list of data association algorithms based on Bayesian probability, such as Joint Probabilistic Data Association Filters (JPDAF) [38] and Multiple Hypotheses Tracking (MHT), recently revisited by [39]. On the other hand, some global association methods are based on the minimization of the cost of matching every tracked identity with the set of detected objects. In [40] a part of the data association process is performed by the Hungarian method [41], where the minimized cost is obtained from the detections locations.

Traditionally, the association task has been improved by means of a filter algorithm, whose purpose is to estimate the state of the tracked objects, especially when a location or motion-based association method is used, like in [42,43]. This last work presents a feature-based approach for detecting and tracking multiple moving targets from UAVs. First, the features are extracted using Harris detector, then the pyramidal Lucas-Kanade (LK) optical flow model, and the Least Median Square Estimator (LMedS) are used in order to classify the movement of the detected features. Finally, a Kalman filter and a template matching algorithm are used to track the detected targets.

The literature presents two main configurations for the application of this type of filters to the MOT task: the decentralized one, which associates a filter to each object, such as the Decentralized Particle Filter (DPF) [44], and the centralized one, where all the objects states constitutes a single representation, like the Tracker Hierarchy [45], and the Reversible Jump Markov Chain Monte Carlo—Particle Filter (RJMCMC) [46].

Moreover, the research has also been focused on enhancing human detection methods. The work presented in [47] employs the position and articulation of the limbs to render each person. Dufek and Murphy presented a pose estimation algorithm of an Unmanned Surface Vehicle (USV) using visual information gathered by a UAV; in order to rescue drowning victims [48]. This system presented a helpful tool to the rescue team, however, it requires parameters tunning, which is not suitable for autonomous tasks.

On the other hand, in the last years, the success of deep Convolutional Neural Networks in some tasks like image classification, [49], and detection [50,51], has inspired a new approach for object tracking. This is based on the leaning of tracking models by means of supervised training. For instance, in [52], it is proposed an algorithm that pre-trains a CNN on a large set of labeled tracking sequences to obtain a generic target representation, and then, online domain-specific learning is performed on new sequences. In this paper, it is proposed the use of models that were pre-trained on different domains, one to detect human joints, and the other to measure the degree of appearance similarity between two images in pedestrians tracking. The lack of labeled rescue sequences makes impossible finetune the models on the target domain. For that reason, they have been used as a partial tool, whose predictions have been properly combined and adapted to the tracking problem on rescue scenarios.

### 2.3. Control

On the other hand, Autonomous UAVs are developed by means of advanced control algorithms in order to perform complex missions. Obstacle detection and avoidance are considered as a critical problem in the autonomous navigation systems, especially in the rescue and search purposes. In these missions, the UAV should be able to perform the task maintaining the distance to the detected objects, as well as avoiding any obstacle appears in front of the UAV. Collision avoidance is achieved by using the information gathered from different sensors. Thus, the first problem to be addressed is how to obtain this information that the avoidance algorithm will have to exploit. Some works are related mainly to the use of the visual information [53], infrared sensors [54], or laser scanners [55].

Watanabe et al. proposed a vision based obstacle avoidance system, where a cone approach is used as criteria to evaluate the danger of impact for the UAV [53]. In this system, the control strategy consists in *Minimizing the Effort in Guidance* (MEG) method for multiple target tracking in a waypoint following the trajectory. The obstacles are detected by radar and their 3D pose is estimated; in order to verify if the collision cone built around their safety boundaries intersects the UAV velocity vector. In case intersection is effectively verified, the obstacle is considered critical to navigation. Obstacles judged to be critical are avoided by changing the aiming point of the UAV by an offset depending on its velocity and, as a result, a collision avoidance maneuver is generated respecting the minimum effort guidance (MEG) method. Nonetheless, this strategy to accomplish obstacle avoidance has been verified only in simulation.

Different strategies based on fuzzy logic to control mobile robots are presented in [56] or, more specifically, UAVs [57,58].

The input for the fuzzy controller is obtained from several different types of sensors. In [57], the data obtained by the ultrasonic sensors are used to measure the distance of external objects, and actuate a path re-planning that takes into account the gathered information. In this approach, the collision avoidance module is built and mounted between the tele-controller (RC) and the flight controller. The inputs of the fuzzy controller are the direction and the distance of the detected obstacles. The fuzzification is performed through the application of three triangular membership functions and, as a result, the following matrices are built: membership grade of obstacle’s direction, membership grade of obstacle’s distance and obstacle’s fuzzy relation matrix between direction and distance. If an obstacle is detected, the obstacle avoidance module will take over the control and the flight-controller will be isolated from the tele-controller, i.e., the user will lose control of the flight during the collision avoidance maneuver.

The strategy has been tested using Matlab and managed to avoid both scattered obstacles and continuous walls. Nonetheless, the loss of control over the UAV during the collision avoidance maneuver represents the main drawback when it comes to implementing an avoidance behavior as a helping feature for the pilot manually controlling the UAV.

Another work based on fuzzy logic to achieve obstacle avoidance is presented in [59]. This work discusses the implementation of an optimized fuzzy visual servoing system. The sensor used is an onboard forward-looking camera, and the visual servoing is achieved through image processing front-end that is used to detect and track obstacles. The detected obstacles generate, through the fuzzy control, a yaw maneuver that makes them shift position in either the left or right edge of the camera field of view and then they are kept there through tracking (that allows the UAV to keep the object always at the same bearing).

The use of a Camshift algorithm [60] allows the extraction of the center of the color region of the obstacle, then it is used to maintain the bearing of the UAV. As a result, the inputs of the fuzzy control are the bearing error, its derivative and its integral, while the output is a yaw correction. The three membership functions are triangular. The cross-entropy (CE) optimization method [61] is used to determine the optimal gains $k_{E}$, $k_{D}$, $k_{I}$ for the three entries of the fuzzy controller. Convergence is achieved when either a cost function is minimized, or the maximum number of iteration is reached. This algorithm has been verified with real flights giving good performances. However, the main drawbacks reside in the high computational power required for the processing that it is thus executed off-board in a ground station.

Other similar approaches to collision avoidance for autonomous systems using fuzzy logic are discussed in [62], that exploits infrared range (IR) sensors to gather information about the environment, and [63] that, conversely, develops a sonar sensor based obstacle avoidance. In the later work, a behavior-based approach, with a hierarchical structure, is presented. The main advantages of such a structure for fuzzy control are: a smaller set of rules, simpler rules (fewer parameters) and reliability and stability easier to test, since each behavior can be verified separately [64].

All the discussed fuzzy logic controllers implement obstacle avoidance as a steering maneuver and regulate their fuzzy sets according to this objective. On the other hand, in semi-automatic control, help is provided to the pilot with the aim of keeping the control intuitive. Consequently, it is desirable that the input of the pilot is not altered with a replanning but, rather, only dangerous components of the velocity towards obstacles are softened or suppressed. In [65], a solution relying on a finite state machine is proposed. The state of the machine are characterized by three critical zones where the UAV can be: *safe zone*, *close zone* and *dangerous zone*. The measurements of the distances to the obstacles are made through a redundant set of ultrasonic sensors, whose data are fused with the IMU measurements in order to discard false measurement due to the UAV rotation. There are two state machines, for roll and pitch, defined over four directions. If the distance measured falls in the *safe zone*, nothing happens. On the other hand, entering the *close zone*, the velocity of the UAV towards the obstacle is progressively diminished according to the distance measurement. When a critical situation is reached, by entering in the *dangerous zone*, a PID controller is activated that makes the UAV converge at a desired distance. This implementation successfully managed to avoid static and dynamic obstacles although the positioning of the ultrasonic sensors was considered not to be optimal. Another solution using a PID controller is presented in [66]. Differently from the previous solution, this is an image-based sensor collision avoidance where two stereo cameras are used to perform depth calculation using color-based tracking. A color-distinct point, that acts as the reference point for image tracking and depth calculation, is created on the obstacle by means of a laser beam generated by an on-board laser transmitter, and the depth measurement is obtained by stereo triangulation. The UAV achieves collision avoidance through position and attitude control with cascade PID controller. The distance and velocity measurements with respect to the obstacle are provided by the vision system and then passed to a hierarchical non-linear controller. The static and dynamic flight test was conducted in a laboratory, which resulted that while at short range the avoidance performance was satisfying at long range the error was unacceptable. The reason is that the accuracy of linear triangulation deteriorates as the distance goes higher. Some strategies, like the multilevel SLAM discussed in [67], allow obtaining fully autonomous flight by building a 3D mapping and estimating the UAV positioning in it. The resulting navigation allows the UAV to efficiently avoid the collision on its own. Nonetheless, it presents a major shortcoming; consisting of the full computation executed off-board because of the high computational power required. Other strategies fully execute on-board the computation necessary to accomplish the task. Gageik et al. [54], proposed a distance controlled collision avoidance implementation, that relies only on ultrasonic and infrared sensors, on which measurements a sensor fusion is applied. In this work, the task is divided into two subtasks, obstacle detection and collision avoidance, which have undergone experimental testing, successfully controlling the UAV distance simultaneously from multiple objects. However, in all the experiments, the UAV operated fully autonomously without manual inputs, thus its usefulness for the semi-automatic control did not verify. The implementation of obstacle avoidance as an assisting feature for a manual controlled UAV, has been discussed in [68,69]. This solution proposed the use of haptic devices to increase the pilot’s awareness of the environment’s dangers through the use of the sense of touch. The control algorithm produces force feedback in the joystick, informing the operator of the upcoming danger. Dynamic Parametric Field and Time to Impact [70] algorithms are only some examples of this kind of approach. Both these strategies exploit distance measurement and UAV velocity to evaluate risk of collisions and feed a tactile actuator with a feedback force, in order to guide the pilot in avoiding the impact. These approaches, however, need the use of particular joysticks for UAV teleoperation; not being exploitable on commonly used RC controllers.

### 2.4. Motivation

With the aim of solving the perception task in search and rescue intelligent systems, the tracking of the target individuals has been addressed, so that they are not missed along the aerial scanning of a wide scenario.

The literature presents a wide variety of tracking algorithms based on predicting objects trajectories. These are unsuitable for the tracking on sequences captured from a UAV, since the point of view is continuously and rapidly changing, and not only its three-dimensional position variates but also its orientation. This causes large variations in the scale, pose, and orientation that the same individual presents even in consecutive frames. For that reason, the matching cost used in this work is not based on the objects locations. Instead of that, a cost value based on the degree of appearance [71] and pose dissimilarity between the newly detected objects and their previously saved representations is proposed.

Moreover, the Convolutional Pose Machine (CPM) presented in [27] is used not only to obtain the objects poses, on which the association method is partially based, but also to filter the detection not corresponding to human beings, in order to avoid the false positives which badly affects the MOT performance.

Due to the lack of labeled rescue sequence, a new probe dataset has been generated for testing our system. However, the generated quantity of data is not enough to train deep models, which would produce over-fitted models. For that reason, the chosen approach consists of applying the outputs of the pre-trained models, as part of a novel data association formulation. This formulation has been specially designed to perform tracking in search and rescue scenarios.

In addition, a semi-autonomous reactive control is presented; to allow the pilot to approach the UAV to the detected body in a safe and smooth maneuver, avoiding any unnecessary catastrophic actions.

## 3. System Overview

This section describes the general architecture of the proposed system, whose main modules are explained in the following sections in detail.

**Figure 1:**
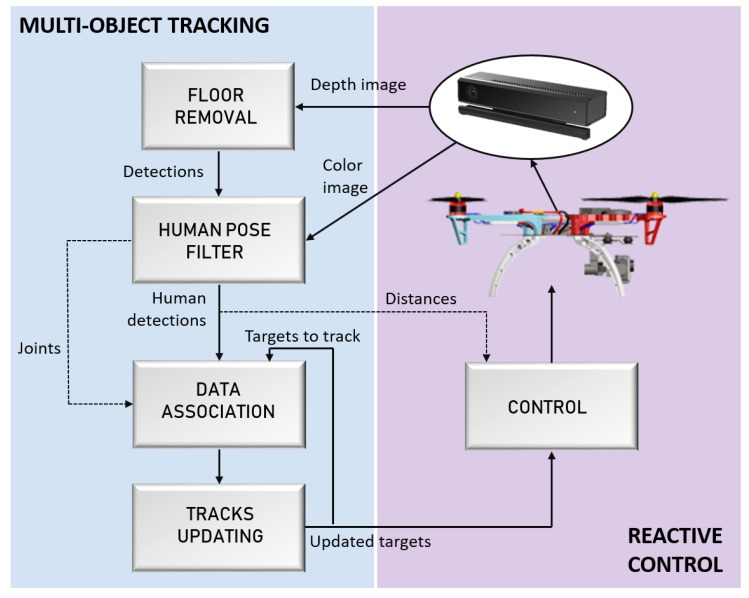
System Overview.

The general architecture of the proposed system is presented by Figure 1, where the stages of the proposed Multi-Object Tracking algorithm (Section 4) are represented in the left column. This algorithm takes the information captured by the sensors placed in the UAV, and gives the location of the found individuals to the reactive control module, which is rendered in the column on the right.

First, the depth image captured by a *Kinect v2* sensor is used to remove the floor surface from a 3D environment representation; in order to extract the existing objects (Section 4.1). The *Kinect v2* sensor is used because it can provide depth information outdoors up to 8 m, which becomes a useful feature for the problem presented in this work. Then, the corresponding regions of interest of these detection are extracted from the color image, which are later analyzed by the human pose filter to discard those not corresponding to human detection, using the human joints detected over a color image (Section 4.2). This way, the human pose filter only has to process the object ROIs, instead of the whole image, thus reducing the processing time.

Subsequently, the data association process assigns every detection with its corresponding identity, taking not only the visual appearance of the human detection in the color image but also their joints locations (Section 4.3). Then, in the next stage, the tracked identities are updated with the data acquired in each new iteration (Section 4.4).

Finally, the information about the updated targets (position and the distances to the UAV) are passed as inputs to the control system; in order to perform approaching maneuvers safely (Section 5).

## 4. Multi-Object Tracking (MOT) Algorithm

This section presents the proposed multi-object tracking (MOT) algorithm, which follows a tracking-by-detection approach. In order to detect the objects on the floor and estimate their sizes, an RGB-D camera is mounted on the UAV pointing downwards. Both, the color image and the point-cloud obtained are used to perceive the environment. The final purpose of the MOT algorithm is to maintain the corresponding identities of the human beings found in the scene through a wide sequence.

**Figure 2:**
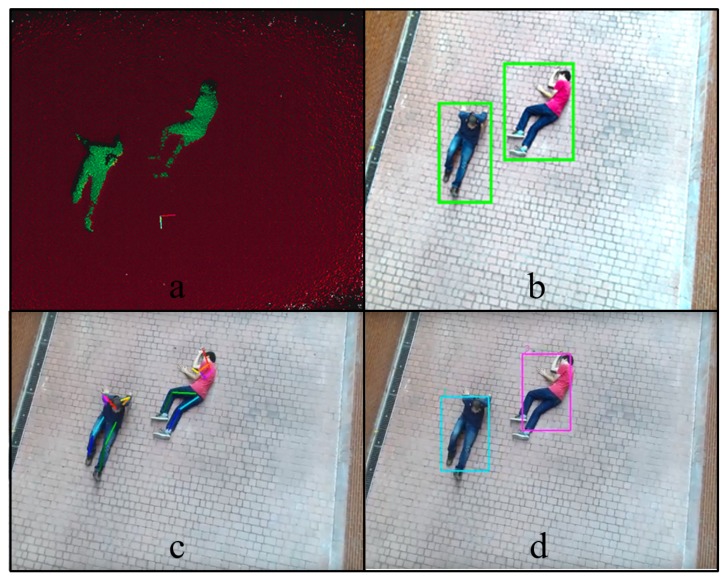
Proposed approach. (**a**) Floor removal stage; (**b**) object detections; (**c**) human pose filter analysis; (**d**) corresponding identity to every human detection.

The proposed approach is divided into four steps, as the blue left part of Figure 1 shows: First, in the floor removal stage, the point-cloud is used to detect the floor plane and extract the outliers points, as Figure 2a presents, and a clustering algorithm is performed over the inliers points to compute their sizes. This first module outputs a set of detections, where humans must be found. Secondly, the object detections with sizes that match target sizes, as presented by Figure 2b, are analyzed by the Human Pose Filter in order to discard false positive detections, using skeleton representations, as Figure 2c shows. Finally, the data association process jointed to the tracks updating step assigns the corresponding identity to every human detection, achieving an online multi-person tracking. An example of the proposed tracking algorithm output for an analyzed frame is presented in Figure 2d, where every identity is rendered by a different color bounding box.

### 4.1. Floor Removal

**Figure 3:**
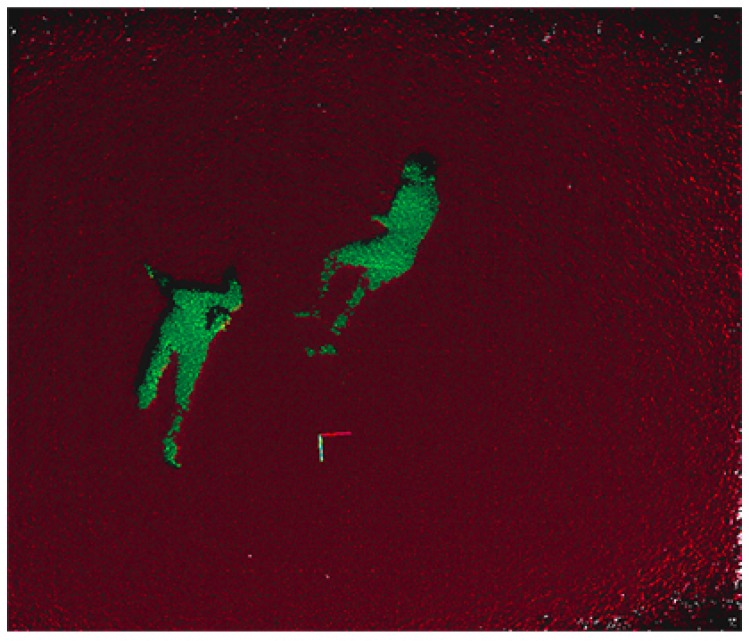
Plane segmentation for floor removal.

During the flight, the 3D information is processed to segment the floor plane and extract the outliers on top of it. For this segmentation method, the RANSAC algorithm is applied to compute the coefficients of the mathematical model of the plane, where the fitted model is defined as a plane perpendicular to the camera. This algorithm relies on the whole floor area, so it is more robust against outliers and works with angled planes. Moreover, this method allows to apply an empirical angle threshold $\epsilon =\pm 15^{\circ }$ and a distance threshold d=±10 cm to estimate the inliers, that increases the robustness of the floor detection, even with inclined planes. Figure 3 shows the point-cloud in a simple scenario with people lying on the floor where the plane segmentation has been performed.

Once the points corresponding to the floor surface have been removed from the point cloud, a clustering algorithm is performed over the rest of the outliers to segment and detect the different objects in the scene. The clustering method implemented is based on the Euclidean distance between points. After that, each cluster is analyzed by its size, removing all objects which size is outside the minimum and maximum boundaries. The values of these boundaries have been experimentally set, accordingly to the usual number of points (for a certain flight distance) that renders a human, which is the object that the UAV is trying to find. This size filtering allows the process to remove small clusters caused by noise and very big objects in the environment , like mounds of debris, which were not filtered by the floor removal.

The regions of interest on the color image, which corresponds to the found 3D object clusters, are extracted, providing a set of detected objects for each frame. Every detection is rendered with an object structure, which is composed by two main attributes, a bounding box defining its location on the color image, and a vector *P*, representing its pose structure. An adapted version of the Convolutional Pose Machine (CPM) presented in [27] has been used to provide this last attribute (The c++ class needed to interpret the pre-trained model and architecture provided by [27] is publicly available under https://github.com/magomezs/CPMclass).

The pose, $P^{o_{i}}$, of and object $o_{i}$ is an array where each element $p_{k}^{o_{i}}$ is a pixel location, $\left(p_{u,k}^{o_{i}},p_{v,k}^{o_{i}}\right)$ of a human joint. The index *k* takes values from 0 to 14, corresponding to these joints, following this order: head, neck, $shoulder_{r}$, $elbow_{r}$, $wrist_{r}$, $shoulder_{l}$, $elbow_{l}$, $wrist_{l}$, $hip_{r}$, $knee_{r}$, $ankle_{r}$, $hip_{l}$, $knee_{l}$, $ankle_{l}$, waist, where the sub-indexes *r* and *l*, stand for right and left, respectively.

The pose of every detection is computed even without the certainty that all of them are rendering human shapes. For that reason, a Human Pose Filter is needed to discard those detections that are not corresponding to people.

### 4.2. Human Pose Filter (HPF)

In order to reduce the number of detected false positives, a filter has been designed to select only those objects presenting a human shape. For the task of rescue, aerial images captured from a UAV are analyzed, so the pose filter must be independent to the rotation and scale. For that reason, an improved version of the shape filter presented in [72] has been designed in order to make it invariant to the rotation and the translation of the viewpoint with respect to the targets to track.

**Figure 4:**
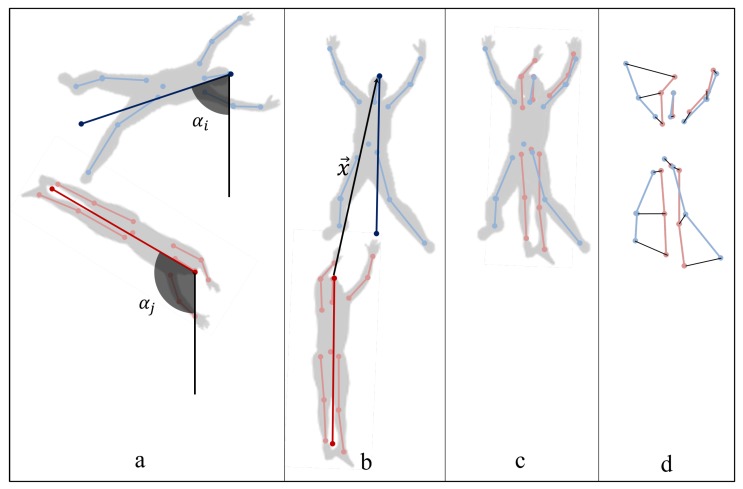
Pose dissimilarity measure $d_{p}\left(t_{i},h_{j}\right)$: (**a**) track, $P^{t_{i}}$, and human object, $P^{h_{j}}$, poses; (**b**) track, $RP^{t_{i}}$, and human object, $RP^{h_{j}}$, rotated poses; (**c**) track rotated pose, $RP^{t_{i}}$, and human object rotated and translated pose, $RTP^{h_{j}}$; (**d**) joints distances.

Consequently, the pose structure of every detected object, $o_{i}$, is rotated around the head (k=0) location according to the Equations (1) and (2), where α is the angle (in radians) needed to obtain a vertical pose. A pose has been considered as vertical when the straight line joining the head location and the average of the feet locations is vertical, as Figure 4 shows.

(1) $$rp_{u,k}=p_{u,0}+\left(p_{u,k}-p_{u,0}\right)\cos\left(\alpha \right)+\left(p_{v,k}-p_{v,0}\right)\sin\left(\alpha \right)$$

(2) $$rp_{v,k}=p_{v,0}-\left(p_{u,k}-p_{u,0}\right)\sin\left(\alpha \right)+\left(p_{v,k}-p_{v,0}\right)\cos\left(\alpha \right)$$

(3) $$\alpha =\frac{\pi }{2}+\arctan\left(\frac{p_{v,0}-\frac{p_{v,10}+p_{v\;13}}{2}}{p_{u,0}-\frac{p_{u,10}+p_{u,13}}{2}}\right)$$

**Figure 5:**
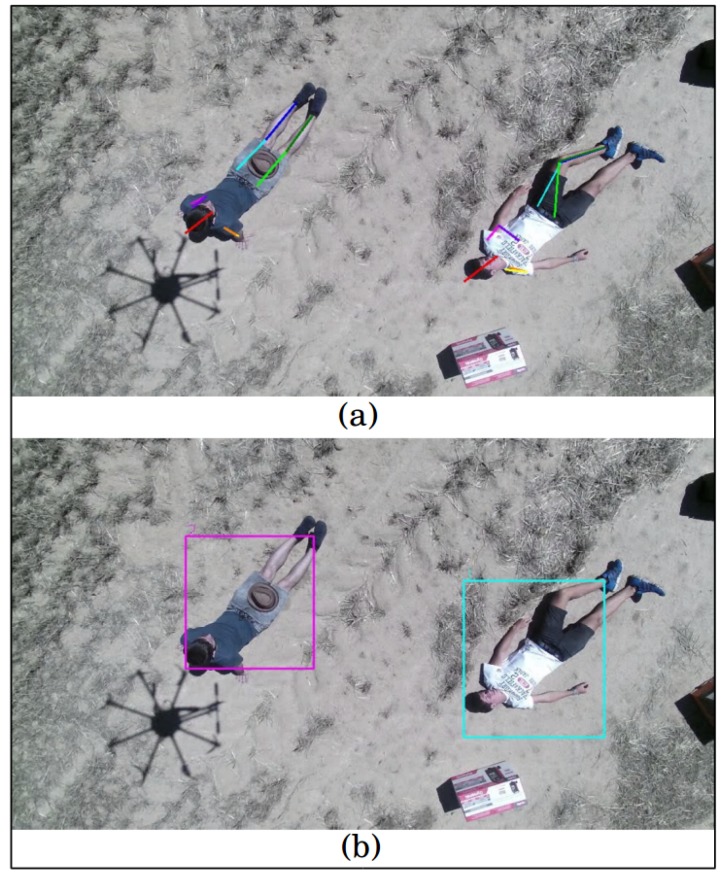
Multi-object tracking (MOT) algorithm output: (**a**) pose structures representation of the detected human objects, (**b**) updated tracks bounding boxes.

Then a set of human objects, *H*, is formed by all those objects, $o_{i}$, whose rotated pose, $RP^{o_{i}}$, satisfies some anatomical constraints, defined in Equation (4), where the function $d_{E}$ is the Euclidean distance, and *T* is a certain threshold to ensure the existence of the head in the human structure. Figure 5 shows the human pose structures found in a frame.

Given the wide range of possible poses that target individuals could present, we have discarded the consideration of symmetry measurements or walking pose detection, which is usually employed in pedestrian tracking domain. Instead of that, the proposed filter formulation considers the presence of a head has a mandatory requirement to denote a detection as human. Subsequently, it discards those poses which are physically impossible from an anatomical point of view, since the poses obtained for non-human detections usually present impossible distributions of the joints locations.

(4) $$\begin{array}{c} H=\{h_{j}:h_{j}=o_{i}|d_{E}\left(rp_{0}^{o_{i}},rp_{1}^{o_{i}}\right)\geq T\wedge \\ \left(rp_{v,0}^{o_{i}}\leq rp_{v,1}^{o_{i}}\right)\wedge \left(rp_{v,1}^{o_{i}}\leq rp_{v,8}^{o_{i}}\right)\wedge \left(rp_{v,1}^{o_{i}}\leq rp_{v,9}^{o_{i}}\right)\\ \wedge \left(rp_{v,1}^{o_{i}}\leq rp_{v,10}^{o_{i}}\right)\wedge \left(rp_{v,1}^{o_{i}}\leq rp_{v,11}^{o_{i}}\right)\} \end{array}$$

### 4.3. Data Association

The data association process is the core of the presented multi-object tracking approach, which is fed by the targets or identities to track, and the previously obtained human detections. The proposed association method is based on the pose and visual appearance. Then, for each tracked identity, it requires an image rendering of the personal appearance, the last time that it was matched and its joints locations in that frame.

Every detected human object must be tracked, so an identification number is associated with each different person appearing in a scene, and its location and pose must be updated at every frame. The set of people tracked, called tracks, is rendered by an array, *T*, of object structures. The matching consists of associating each track with its corresponding human detection in the current frame. A global association approach, based on the Hungarian Method [41] has been adopted. This method finds the assignment between the set of tracks and the set of human detections that optimizes the sum of the matching costs. Therefore, the cost of match every track, $t_{i}$, with every human object, $h_{j}$, must be calculated.

Since the camera location and orientation varies rapidly in the sequences captured from the UAV, the differences between people locations on consecutive images cannot be used as cost. Instead of that, a cost value, *c*, based on the appearance and pose dissimilarities between a track $t_{i}$ and a human object $h_{j}$ is proposed. A modified version of the cost computation presented in [72] has been used, making it invariant to the scale, translation and rotation of the viewpoint with respect to the targets.

The appearance dissimilarity, $d_{a}$, between a track $t_{i}$ and a human object $h_{j}$ is measured as the inverse value of the Degree of Appearance Similarity, DOAS, function, presented in [71], which takes the images rendering both objects as inputs, as it is represented in Equation (6). For a human object, its representation, $R^{h_{j}}$, is the region of the current frame defined by its bounding box, and for the tracks, $R^{t_{i}}$, it was saved in the previous frame.

The pose dissimilarity, $d_{p}$, between a track $t_{i}$ and a human object $h_{j}$ is computed as the weighted mean Euclidean distance between the joints location of the rotated pose structure of the track, $RP^{t_{i}}$, and the rotated and translated pose structure of the human object, $RTP^{h_{j}}$, as it is described by Figure 4, and defined in Equation (7). *W* is a vector, where each element, $w_{k}$, is the weight given to the joint indexed by *k*, and *A* is a vector, where each element, $a_{k}$, takes value 1 if the joint indexed by *k* has been detected in the image and 0, otherwise.

Both pose structures, the one belonging to the query track and the one corresponding to the detected object with which it is being compared, are rotated until a vertical orientation, as it was explained in Section 4.2, and subsequently the rotated pose structure of the human object (in red) is translated the displacement, *x*, needed to align both head locations. In that way, the pose dissimilarity measure is invariant to the differences in the position and orientation of the objects in consecutive images, which is caused by the UAV movements.

DOAS function is given by a classifier, presented in [71] , that was trained on CAVIAR dataset, which is a set of pedestrian tracking sequences. moreover, joints locations are calculated by a pre-trained CPM, given by [27] . None of these models have been fine-tuned on rescue data-sets. The prominent lack of labeled rescue sequences makes impossible to fine-tune these models. Labeled rescue sequences have been generated for testing our system. However, the generated quantity of data is not enough to train deep models on that, which produces over-fitted models. For that reason, the chosen approach consists of directly transferring knowledge from other domains, like pose detection and pedestrian tracking, to the rescue meant algorithm. And consequently, a geometrical adaptation has been made to be able to transfer these different domains models to the rescue task. To achieve that, the different poses that people can present, and the multiple possible camera translation and orientation movements have been considered in our formulation.

(5) $$c\left(t_{i},h_{j}\right)=d_{a}\left(t_{i},h_{j}\right)d_{p}\left(t_{i},h_{j}\right)$$

(6) $$d_{a}\left(t_{i},h_{j}\right)=-DOAS\left(R^{t_{i}},R^{h_{j}}\right)$$

(7) $$d_{p}\left(t_{i},h_{j}\right)=\frac{\sum_{k=1}^{K}d_{E}\left(rp_{k}^{t_{i}},rtp_{k}^{h_{j}}\right)w_{k}a_{k}}{\sum_{k=1}^{K}w_{k}a_{k}}$$

### 4.4. Tracks Updating Strategy

The output of the MOT algorithm is not only the location of the detected people but also their pose and identity for every frame. Once the matching of every track with its corresponding human object detected in the current frame has been done, the object structure of every track is updated, i.e., it is replaced by the object structure of its corresponding human detection, as long as it has been found.

Those tracks whose representation does not appear in a certain frame, are not updated and they keep waiting until being re-identified if they re-appear in the scene.

When a person appears in a frame for the first time, a provisional track is associated with that. In order to avoid false alarms, this type of tracks must be matched with its corresponding detected object in a certain number of frames from its creation, until being considered as a real track, whose updated information is valuable for the rescue task, as it is shown in Figure 5.

## 5. Reactive Control

The ability to sense and approach to the objects (humans) is a crucial feature for UAVs; in order to avoid any catastrophic action that can cause any harm to the human body. For this reason, a semi-automatic control is designed; to aid the pilot of the rescue team to approach the UAV to the detected bodies in a safe and smooth maneuver.

**Figure 6:**
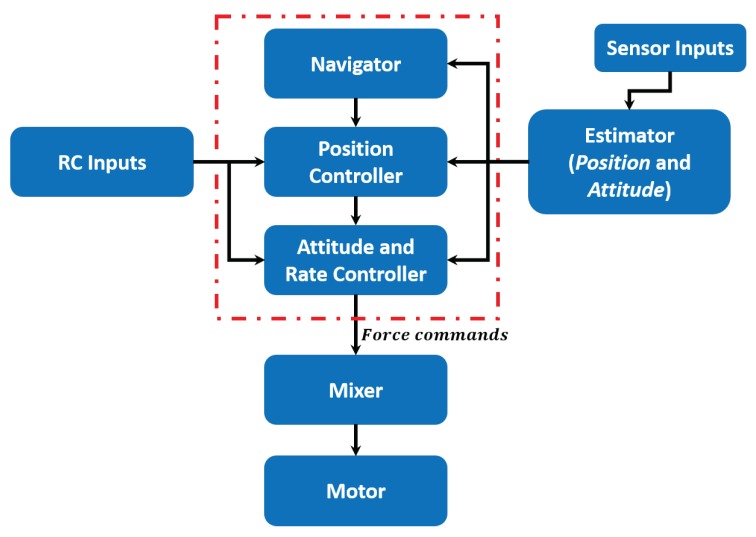
Unmanned aerial vehicle (UAV) Controller loops.

As it is illustrated in Figure 6, the flight controller (Pixhawk) has two controllers: A position and an attitude controller, that allow sweeping different level of controllability going from the *Manual* mode to the fully autonomous *Auto* mode.

### 5.1. Dynamic Model

In order to derive the dynamic model, it is assumed that the UAV is a rigid body, which has 6 Degrees of Freedom (DOF). However, being the UAV controllable only in attitude (Euler Angles or quaternion) and altitude (thrust), the number of controllable DOF reduced to only 4, as a result, the system is underactuated. Moreover, two reference frames are defined; in order to describe the UAV location by its state vectors: North-East-Down (NED) frame and Body frame.

The NED reference frame is a non-inertial frame, centered in the center of gravity of the UAV. Whilst, the body frame represents the orientation of the UAV in the space (Right-hand rule), and has *x*-axis pointing towards the front, while *y*-axis points towards to the right of the UAV and *z*-axis pointing downwards.

The conversion from one frame to the other has been described by a rotation matrix *R* as follows:

(8) $$R=\left(\begin{array}{ccc} c_{\phi }c_{\theta } & c_{\psi }s_{\phi }s_{\theta }-s_{\psi }c_{\phi } & c_{\phi }s_{\theta }c_{\psi }+s_{\phi }s_{\psi }\\ s_{\theta }s_{\psi } & s_{\psi }s_{\phi }s_{\theta }+c_{\phi }c_{\psi } & c_{\phi }s_{\theta }s_{\psi }-s_{\phi }c_{\psi }\\ -s_{\theta } & s_{\phi }c_{\theta } & c_{\phi }c_{\theta } \end{array}\right)$$

where ϕ,θ and ψ are roll, pitch and yaw angles respectively, $c_{\theta }=\cos\theta$ and $s_{\phi }=\cos\phi$, and so on.

Let *x* be the position vector, then $x_{body}=R\cdot x_{ned}$ and $x_{ned}=R^{T}\cdot x_{body}$. The attitude of the UAV is described through Euler angles η, which define the rotations around the body frame axes:

(9) $$\eta =\left(\begin{array}{c} \phi \\ \theta \\ \psi \end{array}\right)$$

(10) $$\dot{\eta }=\left(\begin{array}{c} \dot{\phi }\\ \dot{\theta }\\ \dot{\psi } \end{array}\right)$$

where $\dot{\eta }$ represents the change rate of the Euler angles, which differs from the angular velocity vector ω, which is a vector pointing along the axis of rotation:

(11) $$\omega =\left(\begin{array}{c} \omega_{x}\\ \omega_{y}\\ \omega_{z} \end{array}\right)$$

The conversion from one frame to the other is defined as follows:

(12) $$\omega =\left(\begin{array}{ccc} 1 & 0 & -\sin\theta \\ 0 & \cos\phi & \cos\theta \sin\phi \\ 0 & -\sin\phi & \cos\theta \cos\phi \end{array}\right)\dot{\eta }$$

The UAV is controlled by tuning the speed of rotation of its rotors. Each rotor provides force and torque that are proportional to its rotational speed $\omega_{i}$ measured in rpm (revolutions per minute).

A change in the rpm value causes a variation of the lifting force provided by the corresponding rotor, which consequently causes an alteration of the total lift or of the attitude of the UAV. The total lifting force of the UAV is obtained by summing the single force components generated by each propeller, which is similar for calculating the total torque.

The geometry and dynamics of the UAV are taken into account via some parameters: Arm length *L*, thrust coefficient *k*, the torque coefficient *b*, and the inertia matrix *I*:

(13) $$I=\left(\begin{array}{ccc} I_{xx} & I_{xy} & I_{xz}\\ I_{yx} & I_{yy} & I_{yz}\\ I_{zx} & I_{zy} & I_{zz} \end{array}\right)$$

The total force (thrust) generated by the rotors in the body frame is thus given by:

(14) $$T_{body}=k\left(\begin{array}{c} 0\\ 0\\ \sum \omega_{i}^{2} \end{array}\right)$$

where $\omega_{i}$ is the rpm of a single propeller *i*. Whilst, the total torque in the body frame is modeled as:

(15) $$\tau_{body}=\left(\begin{array}{c} L\cdot k\cdot (\omega_{1}^{2}-\omega_{3}^{2})\\ L\cdot k\cdot (\omega_{2}^{2}-\omega_{4}^{2})\\ b\cdot (\omega_{1}^{2}-\omega_{2}^{2}+\omega_{3}^{2}-\omega_{4}^{2}) \end{array}\right)$$

Thereafter, the aerodynamic effects are described as a force vector:

(16) $$F_{D}=\left(\begin{array}{c} -k_{x}\cdot \dot{x}\\ -k_{y}\cdot \dot{y}\\ -k_{z}\cdot \dot{z} \end{array}\right)$$

In addition, the equation of motion in the inertial frame of the rigid body is obtained as follows:

(17) $$m\ddot{x}=\left(\begin{array}{c} 0\\ 0\\ -mg \end{array}\right)+R\cdot T_{body}+F_{D}$$

where *R* is the rotation matrix as calculated in Equation (8), and the Euler equation for rigid body dynamics:

(18) $$\dot{\omega }=\left(\begin{array}{c} {\dot{\omega }}_{x}\\ {\dot{\omega }}_{y}\\ {\dot{\omega }}_{z} \end{array}\right)=I^{-1}\left(\tau -\omega \left(I\omega \right)\right)$$

#### 5.1.1. Semi-Automatic Control

**Figure 7:**
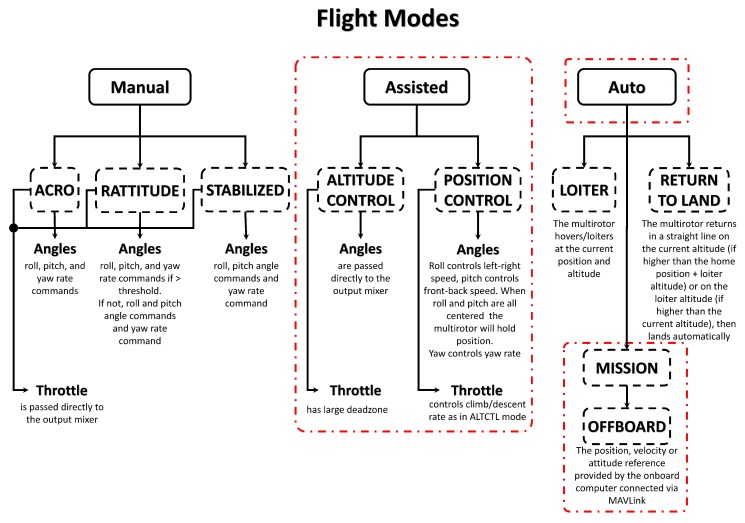
Pixhawk autopilot flight modes.

The proposed semi-automatic control is based on the force feedback, which is obtained by directly modify the reference velocity in higher levels. In order to achieve this, it is important to discuss the architecture of the flight controller (Pixhawk), which provides two control modes (angle and position), whose selection depends on the flight mode chosen. The set of different flight modes are shown in Figure 7.

The *Altitude Control* and the *Position Control* are assisting modes, where the pilot is assisted by the autopilot in the control task. In this work, a semi-automatic obstacle avoidance module is implemented with the *Position Control* flight mode. The correspondent position control mode receives velocity feed-forwards as input. This type of control is highly intuitive sense to different amplitudes of the stick displacement on the RC correspond a proportional change of the quadrotor horizontal velocity.

The semi-automatic control depends on the tunable parameters of the problem, a repulsive velocity field is created and divided into distinct zones, that express different levels of risk in terms of collision and depend on the relative position and velocity with respect to the object.

Let $v_{i}$ be the component of the feed-forward velocity given with the RC in the direction of a detected obstacle, also it is called *approach velocity*. This reference velocity is modified according to the measurement obtained by the RGB-D sensor and the instant velocity of the UAV towards the detected object, then passed down to the position control. Let $v_{Repulsion}$ be the output of the implemented algorithm, the new reference velocity passed to the autopilot will be:

(19) $$v_{Reference}=v_{i}-v_{Repulsion}$$

The advantage in modifying the reference for the position controller is that the feedback loop of the PID controller is exploited to make the UAV converge towards the desired stopping distance, where the repulsive velocity computed by the algorithm equals the approach velocity of the UAV. Since it has been decided to modify the entry of the primary controller, no more modifications are needed. Nonetheless, it is important to remark that, in case it would have been chosen to modify the reference value given as an input in any other controller different from the top one in the software architecture, it would have been necessary to deactivate all the previous controllers, that otherwise would accumulate an error to compensate. Since it is desired to not interfere with the RC inputs if they do not represent a danger for the safety of the UAV, the avoidance procedure is activated only if there is a component of the UAV velocity $v_{body}$ is moving towards an object otherwise the operator input is left unmodified.

**Figure 8:**
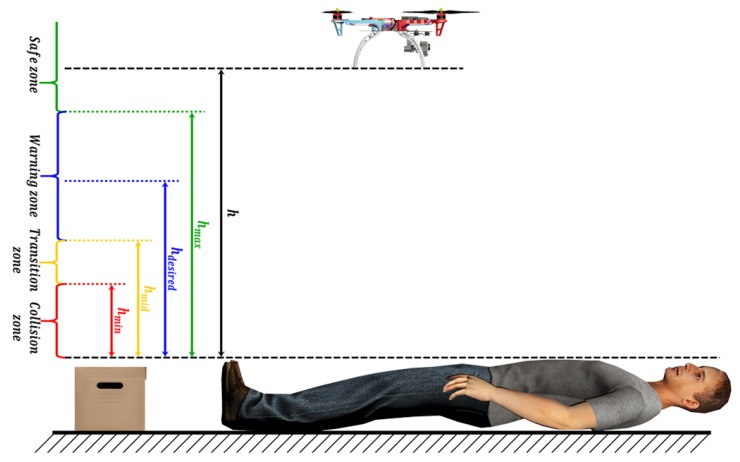
Dynamic Parametric Field algorithm danger zones.

In order to obtain the distance to the detected objects, the 3D information received from the depth sensor is correlated with the color image. Therefore, the actual depth of each pixel in the image is known, so the relative position of the object from the UAV can be computed. This color-depth correlation is performed using the camera intrinsic and extrinsic parameters, as presented in [73]. Let *h* be the distance to the object, then four danger zones are defined as it is shown in Figure 8:

- ***Safe zone***: [$h>h_{max}$], where no risk of impact, thus, no opposing velocity perceived.
- ***Warning zone***: [$h_{max}>h>h_{mid}$], low levels of opposing velocity.
- ***Transition zone***: [$h_{mid}>h>h_{min}$], which makes the pilot aware of imminent impact through a sudden increase in repulsion.
- ***Collision zone***: [$h<h_{min}$], within which the impact is unavoidable.

**Figure 9:**
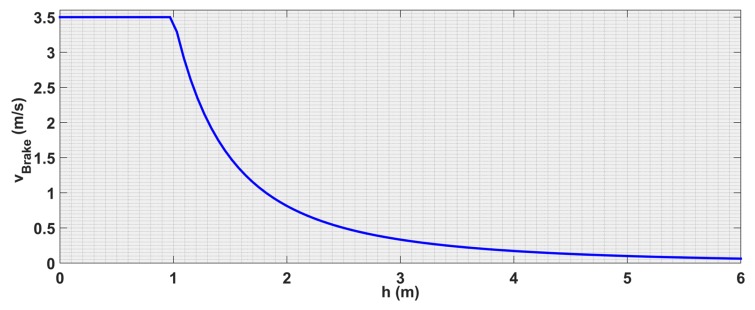
Repulsion curve for $v_{i}=1.5$ m/s and $h_{desired}=1.5$ m.

In this approach, the shape of the repulsive curve is customized to achieve optimal deceleration and parametrized as it is shown in Figure 9, so that its behavior can be easily tuned to the needs of the pilot. There are four parameters that determine the curve shape; the distance at which the UAV should stop $h_{desired}$, the distance at which the maximal velocity feedback is verified $h_{min}$, the maximal velocity feedback $v_{max}$, and the velocity feedback $v_{desired}$ at a distance $h_{desired}$. In addition, $h_{max}$, which defines the starting of the repulsive behavior, and the minimal velocity feedback $v_{min}$.

In this approach, the system is always aware to its surroundings even the UAV locates at safe distances; that is due to the use of the full measurable range, which results that the value of $h_{max}$ is set to the maximum distance measurable by the visual sensor. So that, a sharper change of velocity is provided when the UAV approaches to an obstacle are defined for the curve shape as follows:

(20) $$v_{Repulsion}=\frac{a}{h}+\frac{b}{h^{2}}$$

where, $v_{Repulsion}$ is the output velocity, the parameters *a* and *b* allow to weight the importance of the two components, and are set so that the curve passes through the points $\left(h_{desired},v_{i}\right)$ and $\left(h_{min},v_{max}\right)$, where:

(21) $$\begin{array}{c} a=v_{i}\cdot h_{desired}-\frac{b}{h_{desired}},\\ b=\frac{v_{max}-v_{i}\cdot \frac{h_{desired}}{h_{min}}}{\frac{1}{h_{min}^{2}}-\frac{1}{h_{min}h_{desired}}} \end{array}$$

The choice for the curve to pass by the $\left(h_{desired},v_{i}\right)$ point allows the algorithm to make the UAV to stop at $h_{desired}$. This strategy actuates a simple breaking of the UAV until reaching the desired distance as it is shown in Figure 9.

**Figure 10:**
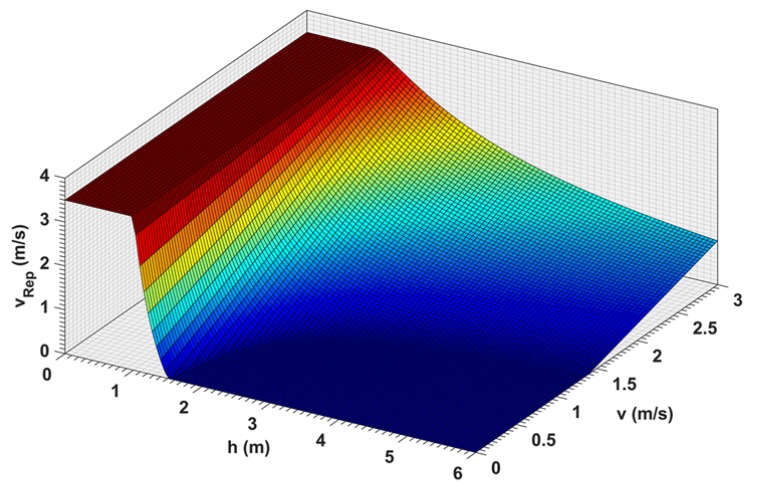
Velocity braking output as a function of velocity and distance.

As a result, the effect on the RC command is the least invasive possible. A repulsion that overcomes the RC command is provided only when this threshold is passed; in order to correct small overshoots due to the time taken by the position controller in converging towards the zero velocity reference. Scaling the curve plot for all the possible approach velocities allows us to obtain the surface plot in Figure 10.

## 6. Experimental Results

Several experiments have been performed; in order to verify the performance and the robustness of the proposed algorithms. For evaluating the MOT algorithm, real flights in outdoor environments have been carried out, taking into consideration different visual conditions. The control approach has been tested in a simulation environment.

### 6.1. Platforms

In the experiments, a carbon fiber octocopter of total weight 4 Kg, based on *Pixhawk 2* autopilot was used. The octocopter is equipped with GNSS, magnetometer and IMU (accelerometers, gyroscopes, and barometer). Furthermore, a down-looking camera *Kinect v2* which provides 1920 × 1080 RGB images, and 512 × 424 infrared patterns, mounted on Walkera G-2D gimbal is used as the main sensor. All the processing is performed on-board by an Intel NUC computer, which has an Intel i7-7567U CPU at 3.5 GHz CPU and 8 GB RAM. The software is integrated with ROS, under Ubuntu operating system.

### 6.2. MOT Results

Both the dataset and the metrics used to evaluate the proposed MOT algorithm are described in the following section, as well as the discussion of the obtained results.

#### 6.2.1. Dataset

In the absence of aerial datasets with available ground truth annotations about the position and identity of multiple human beings for search and rescue purposes, a new dataset has been captured from the mentioned platform (Section 6.1).

The proposed MOT algorithm has been tested over this new dataset, called ARMOT (The ARMOT dataset is publicly available under https://github.com/magomezs/ARMOT-dataset, where the video sequences and its corresponding tracking annotations can be found) dataset, Aerial Rescue Multi-Object Tracking dataset, which is formed by the following four video sequences, listed in order of increasing difficulty:

1. Urban scenario with hovering mode UAV on static human bodies.
2. Urban scenario with hovering mode UAV on dynamic human bodies (walking and jumping), with periods of blurred frames.
3. Open arena with high-velocity flight and static human bodies.
4. Open arena with high-velocity flight with several disappearances and re-appearances of the people in the field of view.

Table 1 presents the video specifications values for every sequence. In addition, every video sequence is accompanied by its corresponding annotations. These consists of the bounding boxes and identities of every human detection at each frame, as long as they are occluded in less than the 50% and their head are visible. This information has been collected in xml files following the format presented by the CAVIAR [74] dataset annotations.

The described sequences form the first version of the ARMOT dataset, which is meant to be enlarged with a wider variety of aerial video sequences.

#### 6.2.2. MOT Metrics

To evaluate the performance of the MOT module, the Multi-Object Tracking Accuracy (MOTA) has been measured as it is defined by Equation (22).

$\bar{F_{N}}$, $\bar{F_{P}}$ and $\bar{Id_{SW}}$ are the summations of the number of false negatives, $F_{N,t}$, false positives, $F_{P,t}$, and identification switches, $I_{s,t}$, respectively, divided by the sum of the number of ground truth objects, $g_{t}$, at every frame, *t*, as Equations (23)–(25) present. Each one of the mentioned tracking errors has been counted according to the definitions presented in [75]. We consider as ground truth object any person whose head and at least 50% of its shape appears in the image.

(22) $$MOTA=1-(\bar{F_{N}}+\bar{F_{P}}+\bar{I_{S}})$$

(23) $$\bar{F_{N}}=\frac{\sum_{t}F_{N,t}}{\sum_{t}g_{t}}$$

(24) $$\bar{F_{P}}=\frac{\sum_{t}F_{P,t}}{\sum_{t}g_{t}}$$

(25) $$\bar{I_{S}}=\frac{\sum_{t}I_{S,t}}{\sum_{t}g_{t}}$$

Table 2 presents the MOT results obtained for the four video sequences (SEQs) of the dataset Section 6.2.1.

Table 2 presents decreasing values of MOTA according to the increase of the difficulties presented by the tested sequences. The decrease of the MOTA value is mainly due to the false negatives rates, which are affected by the large variations of the people visual representation in consecutive frames. These differences are caused by both; the tracked people movements, and the increase in the velocity in the scanning performed by the UAV.

In a rescue task, the velocity with which a large area is scanned is a crucial characteristic, and, even though it causes an increase of the false negatives rate, it does not affect on the real tracking performance. The reason for this is that the presented MOT algorithm is perfectly able to track a target even when some of its detections are missed, and even when the time step between detections is large, thanks to the re-identification ability provided by the proposed degree of appearance similarity comparison. This re-identification success is reflected by the remarkably low rate of identity switches. Moreover, the presented MOT algorithm has been focused on reducing the number of false alarms. Therefore, thanks to the designed Human Pose Filter (HPF), no false positives have been detected or tracked.

### 6.3. Reactive Control Results

**Figure 11:**
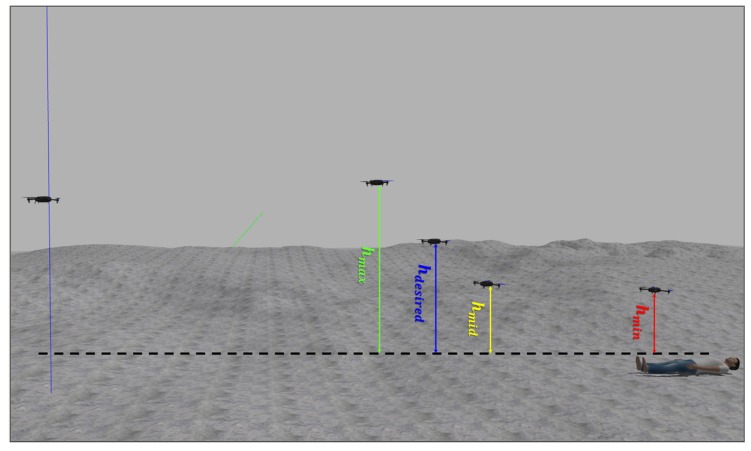
Reference velocity with respect to the distance.

To validate the control presented in this paper, simulation experiments have been carried out, as shown in Figure 11, the proposed control algorithm has been compared with two known strategies; Dynamic Parametric Field (DPF) and Time to Impact (TTI). The simulations consist of three experiments with different velocities, and in the experiments, the input is given at a starting distance to the object greater than $d_{max}$, which allows the UAV to attain the requested velocity before starting the avoidance procedure.

#### 6.3.1. Experiment 1

**Figure 12:**
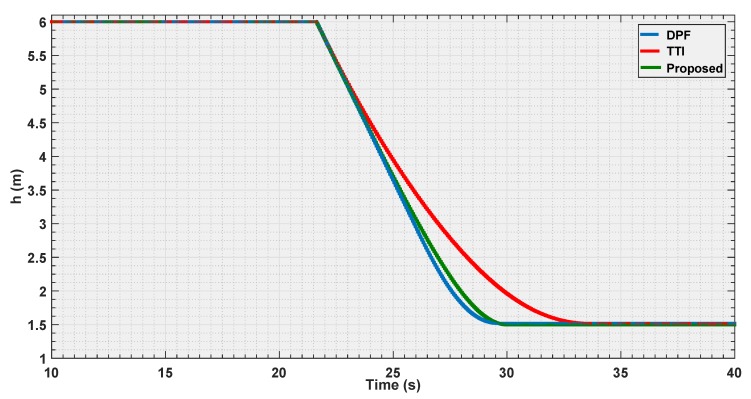
Distance estimation by the three evaluated algorithms *Experiment 1*.

**Figure 13:**
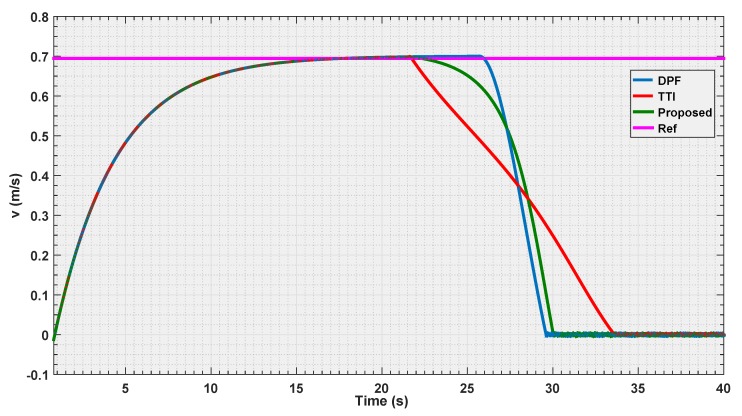
Velocity estimation by the three evaluated algorithms *Experiment 1*.

**Figure 14:**
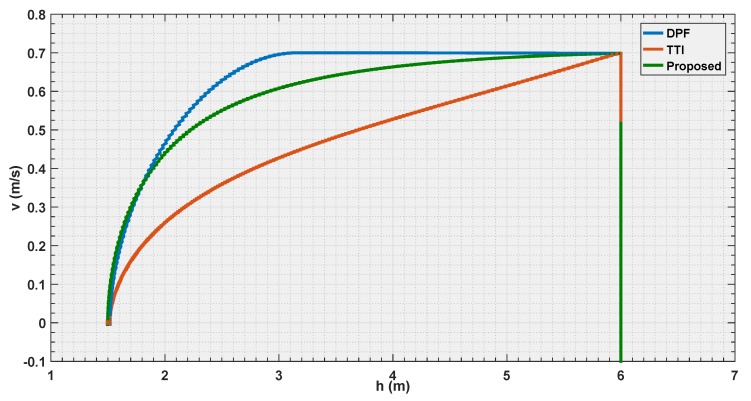
Reference velocity with respect to the distance for the three evaluated algorithms *Experiment 1*.

In the first experiment, the parameters of the three algorithms have been calibrated so that, the UAV flies with velocity of 0.7 m/s, and should stop at the same distance $d_{desired}=1.5$ m from the detected body. Figure 12, Figure 13 and Figure 14 illustrate the evaluation of the three algorithms during the first experiment.

From Figure 12 and Figure 13, it is illustrated that, for the chosen settings, DPF is the first to reach the desired distance, followed by the proposed solution, and finally by TTI that is the slowest to reach $d_{desired}$. The reason for this lies in the distance of activation of the control module. In Figure 14, it is shown that DPF starts the repulsion far closer to the obstacle than the other approaches. Consequently, the deceleration is more uniformly distributed over the avoidance range for TTI followed by the proposed solution that, after an initial, progressively increasing deceleration, reaches a constant steepness zone in the v(t) curve, that means constant deceleration until $d_{desired}$ is reached. The convexity of the curves in Figure 13 depicts a negative jerk. As a result, the curve from the proposed algorithm has the least violent change in velocity at the beginning of the maneuver, oppositely to DPF that, due to the late activation, takes action with a strong jerk at its activation. In the three figures, the proposed parametrized curve shows behavior that falls between the extremities defined by the other approaches, thus representing a good compromise among them.

#### 6.3.2. Experiment 2

The second experiment consists of repeating the previous experience, keeping the same value for the intensity parameters that allowed the vehicle to stop in the three cases, at the same distance to the obstacle of 1.5 m, and observe the effect of doubling the velocity.

**Figure 15:**
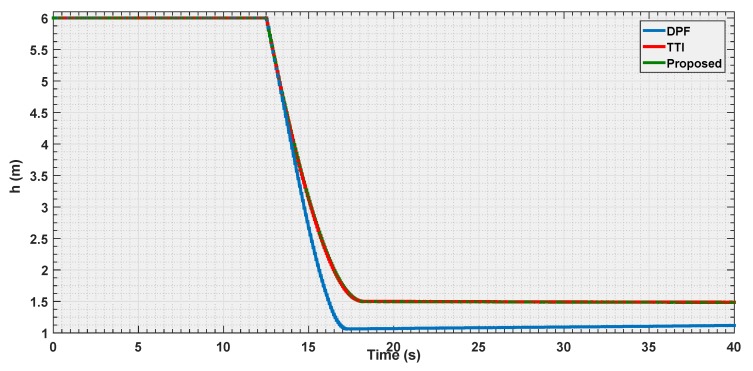
Distance estimation by the three evaluated algorithms *Experiment 2*.

As discussed in the Algorithm implemented section, the TTI and the proposed curve pass through the point $\left(d_{desired},v_{i}\right)$; independently of the input velocity, the UAV will stop at the defined threshold. DPF, on the other hand, does not present this advantage and the previous choice of the parameters causes it to stops just 1 m to the obstacle (see Figure 15).

**Figure 16:**
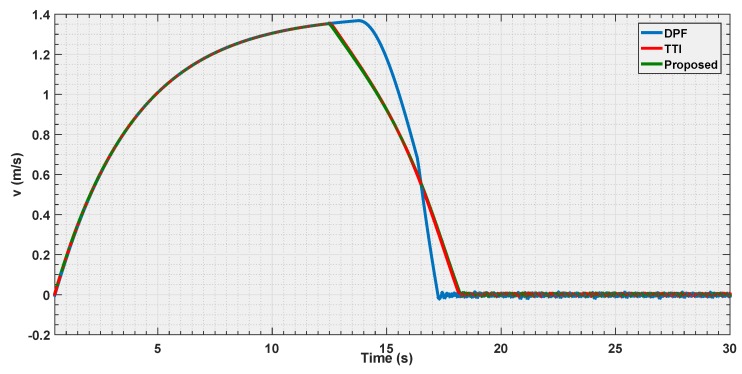
Velocity estimation by the three evaluated algorithms *Experiment 2*.

**Figure 17:**
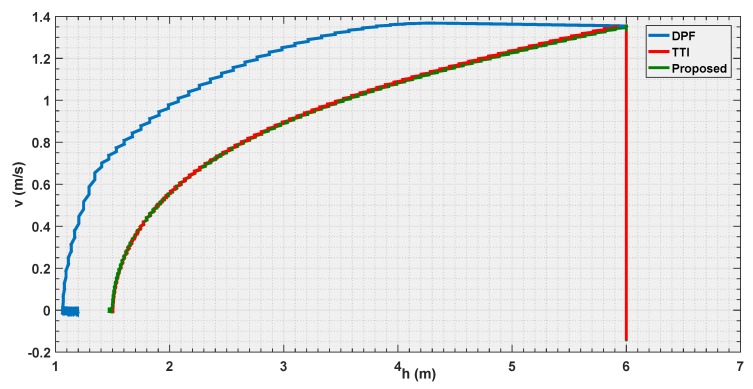
Reference velocity with respect to the distance for the three evaluated algorithms *Experiment 2*.

It is notable how, in the three Figure 15, Figure 16 and Figure 17, the curves for TTI and proposed algorithm are practically coincident. This relates to a peculiar input velocity case, where the *b* coefficient in equation (Equation (20)) is close to 0, making the parametric curve formula collapse onto an hyperbola-like the one mathematically defining the TTI strategy (Equation (19)). Similar to *Experiment 1*, the change of slope in Figure 16 indicates a greater negative jerk produced by the DPF implementation. This is due to the later activation of the collision avoidance maneuver, that thus make DPF the first algorithm to clear the avoidance task even though its trajectory is longer (it stops closer to the obstacle). It could be possible to modify this behavior by setting a higher value, thus allowing an extension of the $d_{max}$ range, and a better distribution of the velocity feedback over the four risk zones, or simply increasing the value of the maximal velocity feedback $v_{max}$.

From Figure 17, it is also evident how the DPF slightly overshoots in its trajectory. This causes the vehicle to reach a distance, where the repulsion is slightly higher than the input velocity leading to a small negative $v_{Reference}$ that makes the UAV to slowly recede.

#### 6.3.3. Experiment 3

The final experiment consists in a repetition of the first one case, where all the strategies are put in condition to make the vehicle stop at the same distance to the obstacle, but using a velocity three times as big as that in the first experiment.

The objective is to observe how the curves shape when the vehicle is approaching its dynamical limits in avoidance.

**Figure 18:**
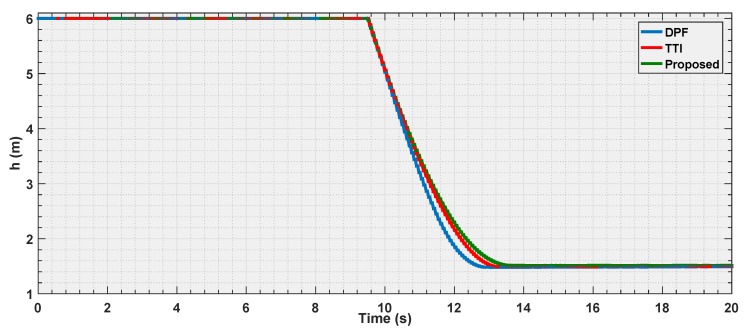
Distance estimation by the three evaluated algorithms *Experiment 3*.

**Figure 19:**
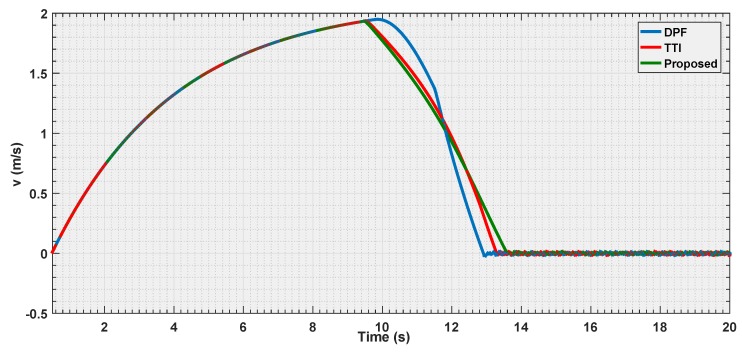
Velocity estimation by the three evaluated algorithms *Experiment 3*.

**Figure 20:**
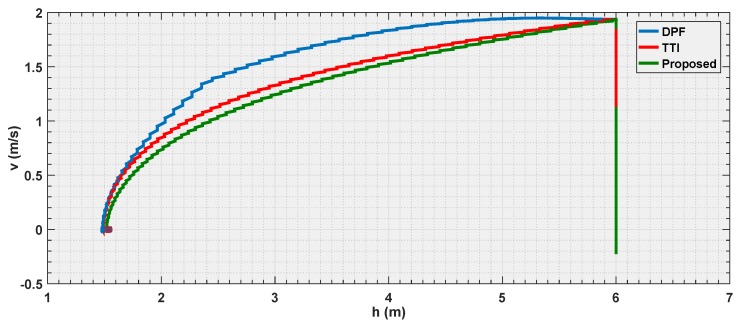
Reference velocity with respect to the distance for the three evaluated algorithms *Experiment 3*.

According to the Figure 18, Figure 19 and Figure 20, it is shown that as the velocity is increased the three tested strategies show similar behaviors, with the three curves almost converging towards the same trajectory. This also shows how the choice of the law (Equation (20)) for the parametric curve is a good pick to describe the increased danger when approaching an obstacle.

It represents an important result since, while the motivation behind the TTI is easy to understand given the fact that, it associates the inverse of the time remaining to the collision to a measure of the danger.

## 7. Conclusions

Search and rescue missions raise special requirements on small aerial robotic systems since it is the process of finding and assisting individuals who are lost in remote wilderness areas.

In this paper, two algorithms have been presented as a framework to cope with cutting-edge UAVs technology in Wilderness Search and Rescue (WiSAR) operations. Multi-Object Tracking algorithm, which provides useful information about the victims in unfriendly environments, and semi-autonomous reactive control, which assists the pilot to perform smooth and safe approaching maneuvers to the detected victims. In addition, in this paper, a new dataset called ARMOT of victims bodies from aerial images has been presented.

The proposed tracking algorithm has been specially designed to cope with aerial sequences, where the appearance, position and orientation of the objects are continuously changing, and the use of the traditional trajectory-based tracking is not viable.

The new association method based on the body pose and appearance allows the performance of the tracking and the identification tasks, in remarkably challenging aerial sequences. In addition, the false positives have been reduced to zero, thanks to the design of the filter based on the Convolutional Pose Machine (CPM).

Furthermore, the proposed parametric curve implementation in the reactive control approach resides in the possibility to select the stopping distance that, if within the dynamic limits of the UAV, will be attended whichever the input velocity given by the pilot.

This framework has been verified by performing real and simulation experiments in urban and open arenas, and the obtained results demonstrate the robustness of the proposed algorithms as a powerful tool to be applied in complex and unconstrained scenarios. 

## Figures and Tables

**Table 1:** ARMOT dataset.

SEQs	Frame Rate (FPS)	Resolution	Length (Frames)	Duration (s)
1	15	960 × 540	221	14
2	30	960 × 540	251	8
3	15	960 × 540	120	8
4	15	960 × 540	645	43

**Table 2:** Multi-object tracking (MOT) metrics values for the sequences 1, 2, 3 and 4.

SEQs	$\sum_{t}g_{t}$	$\sum_{t}F_{N,t}$	$\sum_{t}F_{P,t}$	$\sum_{t}I_{S,t}$	$\bar{F_{N}}$	$\bar{F_{P}}$	$\bar{I_{S}}$	MOTA
1	440	0	0	0	0.000	0.000	0.000	1.000
2	250	73	0	0	0.292	0.000	0.000	0.708
3	12	3	0	1	0.250	0.000	0.083	0.667
4	190	163	0	1	0.858	0.000	0.005	0.137
Total	**892**	**236**	**0**	**2**	**0.265**	**0.000**	**0.002**	**0.733**
